# Developing a highly efficient CGBE base editor in watermelon

**DOI:** 10.1093/hr/uhad155

**Published:** 2023-07-23

**Authors:** Dong Wang, Yani Chen, Tao Zhu, Jie Wang, Man Liu, Shujuan Tian, Jiafa Wang, Li Yuan

**Affiliations:** State Key Laboratory of Crop Stress Biology for Arid Areas, College of Horticulture, Northwest A&F University, Yangling, 712100, Shaanxi, China; State Key Laboratory of Crop Stress Biology for Arid Areas, College of Horticulture, Northwest A&F University, Yangling, 712100, Shaanxi, China; State Key Laboratory of Crop Stress Biology for Arid Areas, College of Horticulture, Northwest A&F University, Yangling, 712100, Shaanxi, China; State Key Laboratory of Crop Stress Biology for Arid Areas, College of Horticulture, Northwest A&F University, Yangling, 712100, Shaanxi, China; State Key Laboratory of Crop Stress Biology for Arid Areas, College of Horticulture, Northwest A&F University, Yangling, 712100, Shaanxi, China; State Key Laboratory of Crop Stress Biology for Arid Areas, College of Horticulture, Northwest A&F University, Yangling, 712100, Shaanxi, China; State Key Laboratory of Crop Stress Biology for Arid Areas, College of Horticulture, Northwest A&F University, Yangling, 712100, Shaanxi, China

## Abstract

Cytosine and adenosine base editors (CBEs and ABEs) are novel genome-editing tools that have been widely utilized in molecular breeding to precisely modify single-nucleotide polymorphisms (SNPs) critical for plant agronomic traits and species evolution. However, conventional BE editors are limited to achieve C-to-T and A-to-G substitutions, respectively. To enhance the applicability of base editing technology in watermelon, we developed an efficient CGBE editor (SCGBE2.0) by removing the uracil glycosylase inhibitor (UGI) unit from the commonly used hA3A-CBE and incorporating the uracil-DNA glycosylase (UNG) component. Seven specific guide RNAs (sgRNAs) targeting five watermelon genes were designed to assess the editing efficiency of SCGBE. The results obtained from stably transformed watermelon plants demonstrated that SCGBE2.0 could efficiently induce C-to-G mutations at positions C5–C9 in 43.2% transgenic plants (with a maximum base conversion efficiency of 46.1%) and C-to-A mutation at position C4 in 23.5% transgenic plants (with a maximum base conversion efficiency of 45.9%). These findings highlight the capability of our integrated SCGBE2.0 editor to achieve C-to-G/A mutations in a site-preferred manner, thus providing an efficient base editing tool for precise base modification and site-directed saturated mutagenesis in watermelon.

## Introduction

Since their discovery, gene editing technologies, particularly CRISPR/Cas9 (Clustered Regularly Interspaced Short Palindromic Repeats/CRISPR associated 9)-based systems, have revolutionized gene function characterization and molecular breeding in plants [1, 2]. The Cas9 protein, guided by sgRNA, targets specific DNA sequences and induces double-strand breaks (DSBs) on-site. Subsequently, cells undergo repair processes, either through non-homologous end joining (NHEJ) or homologous recombination (HR) pathways [3]. Because the CRISPR/Cas system lacks repair templates, it can introduce various modifications to genomic DNA, including indels etc*.* [4].

In crops, single-nucleotide polymorphisms (SNPs) are the critical genetic basis for agronomic traits [5, 6]. Thus, achieving precise and efficient base substitutions is crucial for plant molecular breeding [7], especially with the increasing number of SNPs identified through genomics and pan-genomics that have been found to be essential for agronomic traits and species evolution [8, 9]. To address this need, base editing (BE) systems, based on the CRISPR/Cas9 technology, have been developed for targeted base substitutions. Building upon the advancements in mammalian cell research, optimized cytosine base editors (CBEs) and adenosine base editors (ABEs) have been successfully applied to plant crops. These editors can convert nucleotides without inducing DNA double-strand breaks or requiring donor DNA as a template [10, 11].

The most commonly used BE editors are CBEs and ABEs, responsible for cytosine (C-to-T) and adenine (A-to-G) base substitutions, respectively [12]. A typical CBE consists of cytosine deaminase, nCas9/dCas9 and the UGI. When the CBE fusion protein, guided by sgRNA, targets the target genomic DNA, cytosine deaminase deaminates cytosine (C) to uracil (U) within a specific single-stranded DNA (ssDNA) range determined by the spatial conformation of Cas9 protein, sgRNA, and genomic DNA. Subsequently, during DNA repair and DNA replication, uracil is converted to thymine (T), resulting in C-to-T base substitutions [13, 14]. In 2016, the first-generation cytosine base editor BE1 (rAPOBEC1-dCas9) was developed in David Liu’s lab. Subsequent improvements led to the development of BE3 (rAPOBEC1-nCas9-UGI), which significantly enhanced base editing efficiency but often generated unnecessary indels during the editing process [14]. Further advancements have been achieved with the discovery of new cytosine deaminases, such as PmCDA1, hAID, and hAPOBEC3A, resulting in improvements in base editing efficiency, editing window, and reduction of indels frequency [15–17]. Consequently, multiple deaminase-based CBEs have been extensively employed in various plant species, including monocotyledonous crops like rice, maize, and wheat [18–20], as well as dicotyledonous crops like cotton, tomato, and watermelon [21–24]. These advancements provide powerful tools for efficient and precise base editing in plants. Notably, BE3 based CBE successfully achieved an amino acid substitution (P190S) in the herbicide resistance gene *ClALS* in watermelon (*Citrullus lanatus*), resulting in the development of the first single-base edited herbicide-resistant watermelon variety. The watermelon CBE attained an editing efficiency of 23.0% in the T0 generation, marking a significant milestone for watermelon BE editor development [24].

ABE, similar in structure to CBE, operates through a comparable mechanism. Adenosine deaminase converts adenine (A) to inosine (I), which is subsequently recognized as guanine (G) by DNA polymerase during DNA repair and replication [12, 13, 25]. Initially, the natural adenine deaminase could not utilize DNA as a substrate for base deamination. However, the deaminase suitable for ABE was artificially evolved from the *Escherichia coli* tRNA-specific adenosine deaminase (ecTadA). The widely adopted ABE version is 7.10 (ecTadA-ecTadA*-nCas9) [26], but recent optimizations have resulted in the development of ABE8e (ecTadA8e-nCas9). This improved version exhibits significantly enhanced base editing efficiency and maintains a consistent editing window during the editing process [27, 28]. To date, ABE has been successfully applied in several plant species, including rice, cotton, *Arabidopsis*, and poplar [29–33].

The optimization of BEs has primarily focused on enhancing base editing efficiency, modifying the editing window as needed, and reduction of protospacer-adjacent motif (PAM) sequence dependence [34, 35]. Improvements have been achieved through the utilization of different types of deaminases or the introduction of key amino acid mutations in existing deaminases, such as hAPOBEC3B, TadA8e (V106W), and TadA9 [27, 36, 37]. These modifications allow for optimization of both the base editing efficiency and the editing window. Furthermore, the use of Cas protein variants, including Cpf1, SpCas9-NG, and SPRY, has significantly expanded the range of selectable PAM sequences [38–40]. These optimizations in various components empower BE editors to become more efficient and versatile tools for plant research and breeding applications.

Although C-to-T and A-to-G base substitutions are well established in various plant species through CBEs and ABEs, their limited capability significantly restricts the application of base editing technology in crop molecular breeding [41]. UNG is a widely conserved enzyme found in both plant and animal cells. It plays a crucial role in the initiation of base excision repair (BER) by removing uracil from the DNA double-strand [42, 43]. Recent studies have shown that CGBE (C-to-G base editor) can enhance C-to-G editing in mammalian cells by either eliminating UGI from CBE or replacing it with UNG [43–45]. In rice plants, the newly developed OsCGBE03 (Anc689 (R33A)-CGBE) achieved efficient conversion of cytosine to guanine at the target sites, with an average frequency of 21.3%, significantly expanding the editing potential of traditional CBE [41, 46]. Additionally, in poplar, CGBE has been established with rAPOBEC1 (R33A) deaminase, but it only exhibited a monoallelic editing efficiency of 6.25% in T0 lines [47]. In the case of rice, another CGBE editor composed of evoFENRY deaminase, nCas9NG, and UNGs (human UNG and rice UNG) was tested, revealing that the average C-to-G editing efficiency without indels was slightly higher for CGBE-hUNG (8.2%) compared to CGBE-rUNG (5.9%) in T0 lines [48]. Moreover, the N46L mutation of the TadA8e enzyme offers enhanced cytosine deamination capability with higher C-to-G editing efficiency and a narrower editing window, although further validation is required before its application in plants [49]. In any case, CGBE significantly expands the possibilities of base editing, generating a broader range of base substitution types in crops.

Watermelon, a highly popular summer fruit globally, is renowned for its refreshing taste and abundant nutritional value. Previous studies on the watermelon genome and genome-wide association study (GWAS) analysis have systematically elucidated the molecular mechanisms underlying trait evolution [50]. These findings suggest that SNP substitutions may play a role in the development of traits such as flesh color and sugar content [51, 52]. Expanding the repertoire of base editors beyond CBE holds great potential for advancing molecular breeding in watermelon. Moreover, the progress made in other plant species has inspired the development of an efficient CGBE specifically tailored for watermelon. Therefore, in this study, we have successfully established the first CGBE editing system in watermelon. Our findings demonstrate that the utilization of human APOBEC3A (hA3A) deaminase and *Arabidopsis* UNG enables highly efficient C-to-G/A mutations in a site-preferred manner. This breakthrough opens up new possibilities for molecular breeding and artificial evolution not only in watermelon but also in other dicotyledonous plants.

## Results

### hA3A-CBEs induce C-to-G base substitutions alongside C-to-T editing in watermelon

**Figure 1:**
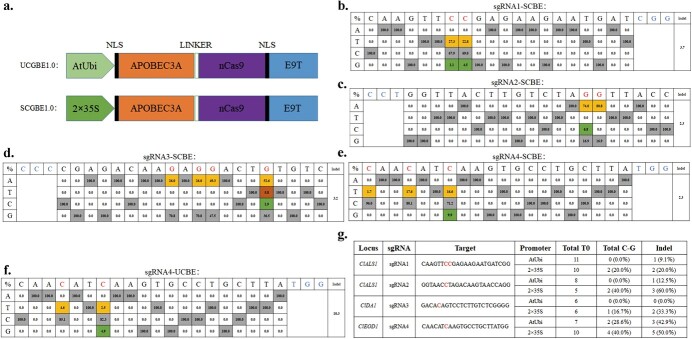
Efficient cytosine base editing mediated by nCas9-fused hA3A in watermelon. **a** Illustration of the hA3A-CBEs constructs employed in watermelon. **b–e** Summary of nucleotide changes for sgRNA1 (**b**), sgRNA2 (**c**), sgRNA3 (**d**), and sgRNA4 (**e**) resulting from SCBE in watermelon T0 lines. Nucleotide changes and the PAM sequences are highlighted. **f** Deep sequencing results for sgRNA4 obtained using UCBE in watermelon T0 lines. **g** Genotyping results of T0 plants edited by UCBE and SCBE**.**

The discovery of hA3A deaminase has revolutionized CBEs by expanding their editing windows and enhancing base editing efficiency [53, 54]. This breakthrough has introduced a more suitable single-base editing tool for studying saturation mutations in target genes through artificial evolution. Furthermore, studies have demonstrated that the choice of promoter can significantly impact the activity of base editing systems in dicot plants [33, 55, 56]. In addition to the commonly used Ubi promoter in plant base editing studies [32, 57], we selected the 35 s promoter, which has been previously used in watermelon CBE experiments, for comparison. Furthermore, in *Arabidopsis* BE studies, it was observed that CBE and ABE carrying the Ribosomal protein S5a (Rps5a) promoter, which exhibits activity in dividing cells, achieved efficient editing successfully [33, 56]. Therefore, in our pursuit of optimizing CBE for watermelon, we aimed to investigate whether the AtRps5a promoter would also impact CBE in watermelon. To this end, we employed various promoters, namely AtUbi (UCBE), 2 × 35S (SCBE), and AtRps5a, to drive the expression of fusion proteins composed of *Arabidopsis* codon-optimized hA3A-nCas9 (D10A)-UGI (Fig. 1a).

To evaluate the base editing efficiency of CBEs, four sgRNAs targeting three watermelon genes (*ClALS1*, *ClDA1*, and *ClEOD1*) were designed, and multiple transgenic watermelon plants were generated. The results revealed that both the AtUbi and 2 × 35S promoter-based CBEs achieved C-to-T editing in watermelon, with the highest base conversion efficiencies (base conversion reads/total reads) recorded as 27.0% and 93.4%, respectively (Fig. S1, see online supplementary material). Interestingly, consistent with previous observations [53], some samples exhibited C-to-G transversions in a few targets alongside the dominant C-to-T edits. This occurrence is likely due to the recognition and excision of C-to-U mutations by endogenous UNG, resulting in abasic sites that initiate DNA repair processes and randomly generate C-to-G/A or indel mutations [13].

We observed C-to-G mutations induced by SCBE in all four target sites (Fig. 1b–e; Fig.S2, see online supplementary material). Among the 31 T0 plants, nine plants demonstrated successful C-to-G substitutions (Fig. 1g), resulting in an editing efficiency of 29.0% (9/31, edited plants/total T0 transgenic plants). In contrast, T0 plants utilizing UCBE alone yielded only 2 C-to-G edited plants in target 4 (Fig. 1f; Fig.S2, see online supplementary material). This suggests that the 2 × 35S promoter is more effective in promoting C-to-G editing compared to the AtUbi promoter in hA3A-CBE. The C-to-G base conversion efficiencies in SCBE and UCBE reached 9.9% and 4.9%, respectively (Fig. 1e and f), which were relatively lower compared to C-to-T mutation frequencies. Conversely, the AtRps5a promoter did not demonstrate any editing evidence. While a previously reported watermelon CBE with BE3 successfully achieved C-to-T editing in the *ClALS1* gene [24], its capability for C-to-G editing remains uncertain. We speculate that the higher occurrence of C-to-G transversions in our CBEs may be a collateral effect of hA3A deaminase in watermelon. These results have motivated us to further optimize the C-to-G editing capacity of hA3A-CBEs in watermelon.

### Removing UGI from CBEs greatly increased C-to-G base conversion efficiency in watermelon

**Figure 2:**
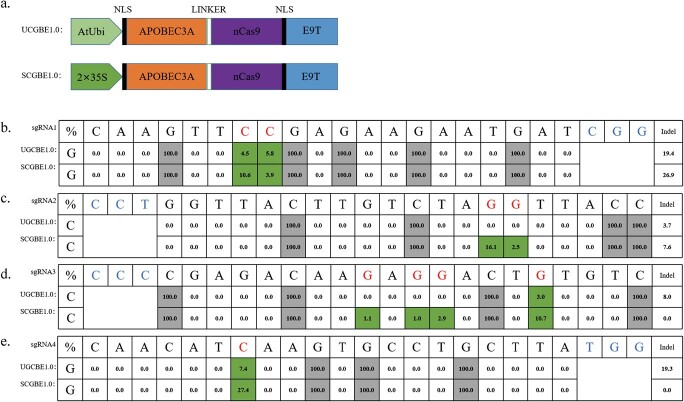
**.** C-to-G base editing mediated by UCGBE1.0 and SCGBE1.0 in watermelon. **a** Illustration of the CGBE1.0 vectors employed for base editing. **b–e** Representative deep sequencing results of four sgRNAs: sgRNA1 (**b**), sgRNA2 (**c**), sgRNA3 (**d**), and sgRNA4 (**e**) generated by CGBE1.0 s in watermelon T0 lines. The editing ratios of C-to-G and the PAM sequences are highlighted.

In CBE systems, the U deaminated from C can be recognized by the endogenous UNG protein present in the cell. UNG cleaves to the glycosidic bond between uracil and the deoxyribose backbone. However, the presence of UGI stabilizes the C-to-T editing by inhibiting the activity of UNG [13, 41]. Theoretically, removing UGI from CBE editors may increase the likelihood of C-to-G transversions. To address this, we eliminated UGI from our CBE systems, resulting in the development of two new editors: UCGBE1.0 and SCGBE1.0 (Fig. 2a).

To further investigate the editing potential of CGBE1.0 in watermelon, stably transformed watermelon plants were utilized, employing the same four sgRNAs as in the CBEs. The Hi-TOM deep sequencing results (Fig. 2b) revealed that UCGBE1.0 achieved C-to-G editing in three targets (sgRNA1, sgRNA3, and sgRNA4) compared to UCBE (sgRNA4), but SCGBE1.0 exhibited higher efficiency in inducing C-to-G transversions across all four targets (Fig. 2b–e). Notably, at the C7 position of sgRNA4, SCGBE1.0 achieved a C-to-G conversion efficiency of up to 27.4%, surpassing UCGBE1.0, which achieved 7.4% efficiency (Fig. 2e). These findings indicate that the 35S promoter-driven SCGBE1.0 is more effective in inducing C-to-G mutations compared to UCGBE1.0. Consequently, SCGBE1.0 was selected for further optimization.

### The addition of UNG has further increased the C-to-G editing capacity of the SCGBE editor.

**Figure 3:**
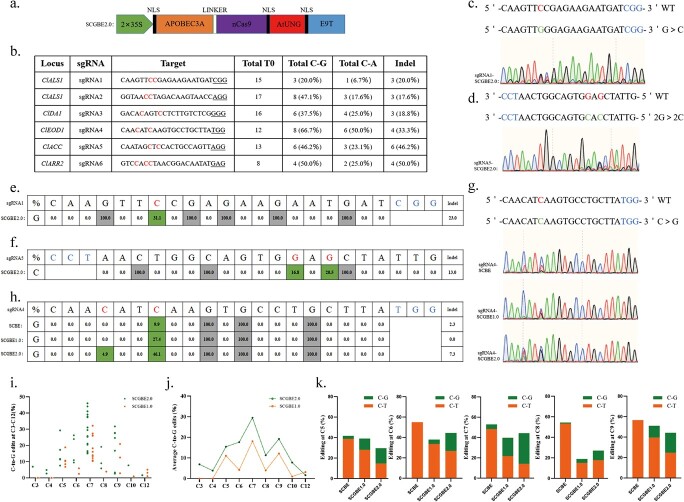
Development of SCGBE2.0 editor in watermelon. **a** Schematic illustration of the SCGBE2.0. **b** Genotyping results of T0 plants edited using SCGBE2.0. **c–d** Sanger sequencing chromatograms of sgRNA1 (**c**) and sgRNA5 (**d**) displaying editing events. Nucleotide changes and the PAM sequences are highlighted. **e–f** Deep sequencing results showing nucleotide changes caused by SCGBE2.0 in sgRNA1 (**e**) and sgRNA5 (**f**) in watermelon T0 lines. The editing ratios of C-to-G and the PAM sequences are highlighted. **g** Typical examples of sequence chromatograms showing C-to-G base editing in sgRNA4 using SCBE, SCGBE1.0, and SCGBE2.0. **h** Comparison of deep sequencing results for C-to-G base editing in sgRNA4 using SCBE, SCGBE1.0, and SCGBE2.0. **i** Dot graph illustrating the C-to-G conversion frequencies of SCGBE1.0 and SCGBE2.0 at positions C3–C12. Each data point represents one biological replicate at each target site. **j** Line graph showing the average C-to-G conversion efficiencies of SCGBE1.0 and SCGBE2.0. **k** Comparison of the C-to-T/G conversion fractions of all targets of SCBE, SCGBE1.0, and SCGBE2.0 at positions C5–C9.

To enhance the editing performance of CGBE, the *Arabidopsis* UNG (AtUNG) was combined with SCGBE1.0, resulting in the development of a new editor called SCGBE2.0 (Fig. 3a) [42, 43, 46]. In addition to the previously tested four sgRNAs, two additional targets (sgRNA5 and sgRNA6) were introduced, with PAM sequences NGG and NAG, respectively, targeting two genes (*ClACC* and *ClARR2*). A total of 81 T0 plants were obtained, and Hi-TOM deep sequencing analysis was performed. The results showed that 35 lines of these plants achieved C-to-G substitutions, representing an editing efficiency of 43.2% (35/81), and the highest efficiency is up to 66.7% (8/12) in watermelon T0 lines (Fig. 3b).

The Sanger sequencing results of sgRNA1 and sgRNA5 demonstrated the effectiveness of SCGBE2.0 in achieving efficient C-to-G base substitutions with indels (Fig. 3c and d), the actual efficiency at position C7 of sgRNA1 was 31.1% (Fig. 3e), and for sgRNA5 it was 20.5% (Fig. 3f), while other C sites exhibited lower or no editing compared to C7. Furthermore, the Sanger sequencing chromatograms of SCBE, SCGBE1.0, and SCGBE2.0 were compared using sgRNA4 as a representative example of our optimization efforts (Fig. 3g). The results clearly demonstrated the superior performance of SCGBE2.0, with the actual C-to-G conversion reads at C7 increasing from 9.9% (SCBE) to 46.1% (SCGBE2.0) (Fig. 3h). Although our sequencing data suggest that SCGBE2.0 may prefer efficient editing at position C7, a few exceptional samples were also observed. For instance, one sample (sgRNA5) exhibited an 80.2% conversion reads at C9, with no editing observed at other C sites (Fig. S3, see online supplementary material). However, this particular sample was excluded from the statistical analysis due to its specificity.

To facilitate the application of CGBE in watermelon molecular breeding, it is essential to futher determine the main editing windows of SCGBEs. Because no C-to-G edits were observed at C loci other than C3-C12 in SCGBE2.0, the actual editing data for edited C loci were analysed (Fig. 3i), SCGBE2.0 demonstrated higher conversion efficiencies at nearly all target sites compared to SCGBE1.0, and the most efficient editing was achieved at position C7, reaching 46.1% (Fig. 3g and h). Furthermore, the average C-to-G base conversion efficiencies higher than 10% were considered valid edits, thus the analysis results revealed that SCGBE2.0 could achieve valid C-to-G substitutions at positions C5-C9 (Fig. 3j), and the average conversion efficiencies ranged from 11.3% to 30.0%, with the highest average editing efficiency observed at position C7 (30.0%). These results provide clear evidence of SCGBE2.0’s broad range of effective editing capabilities and its remarkable efficiency in converting C-to-G bases, particularly at position C7.

Further analysis revealed that 29.6% (24/81) of the T0 plants exhibited both C-to-T and C-to-G edits at the same position when SCGBE2.0 was used. To gain a better understanding of the increased efficiency of C-to-G editing in SCGBE2.0, a comparison of the actual C-to-T and C-to-G conversion fractions at positions C5–C9 was conducted among SCBE, SCGBE1.0, and SCGBE2.0 (Fig. 3k). The results indicated that the total C-to-G/T mutation rates were highest in SCBE, with C-to-T mutations being the dominant type (Fig. 3k). This may be attributed to the UGI component stabilizing C-to-T mutations and reducing the likelihood of other mutations in SCBE [58]. However, both SCGBE1.0 and SCGBE2.0 exhibited a higher C-to-G base conversion ratio within the total C-to-T/G mutations, particularly at the C7 position. Additionally, SCGBE2.0 demonstrated more advantages when compared to SCGBE1.0 (Fig. 3k), supporting the notion that SCGBE2.0 achieved the highest actual C-to-G conversion efficiency (Fig. 3i and j). The results unequivocally showcase the remarkable optimization of CGBE achieved by eliminating the UGI unit and incorporating the UNG component in watermelon.

### SCGBE2.0 achieved efficient C-to-A editing at the position C4 of the targets

**Figure 4:**
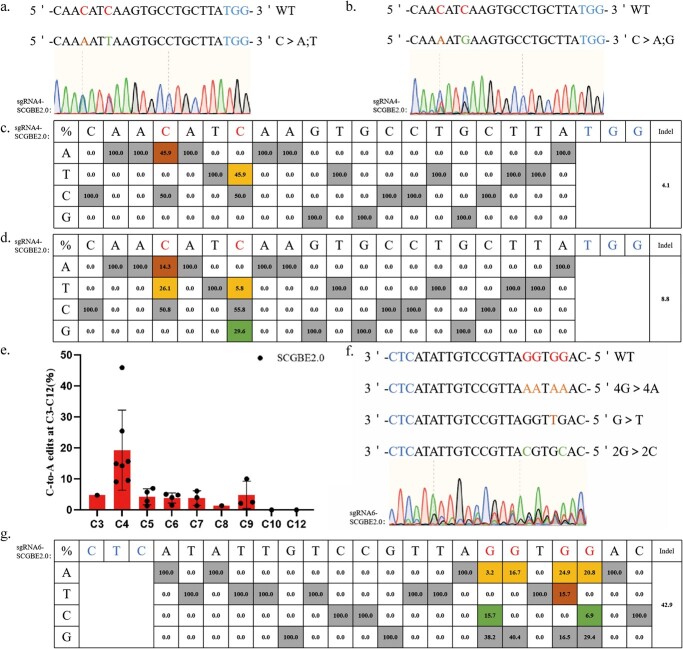
Main C-to-A mutation site and PAM preference in SCGBE2.0. **a–b** Examples of Sanger sequencing chromatograms displaying C-to-A editing at position C4 in sgRNA4. **c–d** Deep sequencing results at position C4 in sgRNA4. The edited bases and the PAM sequences are highlighted. **e** Dot plot illustrating the C-to-A editing frequencies of SCGBE2.0 at C3–C12 sites. Each data point represents one biological replicate at each target site. **f** Typical example of Sanger sequencing chromatograms demonstrating C-to-G/A editing by SCGBE2.0 in sgRNA6 when the PAM is NAG. **g** Actual deep sequencing results for sgRNA6.

According to our results, the novel hA3A-based CGBE editors successfully achieved C-to-G base substitutions. However, additional C-to-A mutations also occurred at specific positions. A total of 23.5% (19/81) of the transformants containing C-to-A mutations (Fig. 3b), primarily concentrated at position C4 (Fig. 4a and b). In Figure 4a and4c (sgRNA4), the predominant editing forms in this sample were C-to-A at position C4 (45.9%) and C-to-T at position C7, with 45.9% representing the highest C-to-A base conversion efficiency among all analysed samples. On the other hand, in Fig. 4b and d (sgRNA4), the predominant editing forms in the sample were C-to-A at position C4 (14.3%) and C-to-G at position C7. These findings suggest that C-to-A editing at the C4 position may occur independently of other forms of C-site editing. Further analysis was conducted to examine the C-to-A mutation pattern of SCGBE2.0 in detail, revealing that efficient C-to-A substitutions were not achieved at sites other than C4, with the editing efficiency generally remaining below 10% (Fig. 4e). Notably, C-to-A mutations were achieved more efficiently than C-to-G mutations at position C4, likely due to the fact that C4 is more suitable for mutation of C-to-A editing in hA3A-CGBE.

Meanwhile, a brief test was conducted to examine the PAM preference of SCGBE2.0. The results demonstrated that SCGBE2.0 successfully achieved C-to-G mutations at position C7 (15.7%) and C-to-A mutations at position C4 (15.7%) when the NAG PAM was used (sgRNA6) (Fig. 4f and g). However, no edits were detected when sgRNA7 with the NGA PAM was employed (Fig. S4, see online supplementary material). These findings indicate that the final integrated SCGBE2.0 editor prefers NGG and NAG PAM sequences over NGA PAM, allowing for site-preferred C-to-G/A conversions and providing more possibilities for the application of SCGBE2.0 in watermelon.

## Discussion

### The development of CGBE opens up new possibilities for watermelon breeding research

Base editors, including CBE for C-to-T mutations and ABE for A-to-G mutations, have emerged as powerful gene editing tools based on CRISPR/Cas9 [12]. These tools enable precise base substitutions at target sites, thereby facilitating agronomic trait improvement. However, the fixed base mutation types offered by CBE and ABE limit their application in crops. Furthermore, it is important to acknowledge the limitations of CBE and ABE, which can only achieve a single type of base substitution within the editing window. To overcome this limitation, researchers have developed a dual-base editor by combining cytosine and adenosine deaminases into a single vector [57]. This groundbreaking innovation allows for the simultaneous editing of C-to-T and A-to-G within the target site. As a result, the introduction of CGBE is seen as a valuable enhancement to the dual base editor, as its ability to edit C-to-G/A is particularly relevant for studying artificial evolution in plants through saturation mutation. Therefore, the development of CGBE editors with C-to-G/A mutation capabilities in watermelon is highly significant.

While high-efficiency C-to-G editors have been established in rice, the optimization of poplar CGBE editors based on rAPOBEC1 is still needed due to their low editing efficiency in poplar T0 lines [46, 47]. In order to better utilize CGBE in watermelon and other dicotyledonous plants, various optimizations have been carried out specifically for watermelon CGBE in present study. Our data demonstrate that the development and optimization of hA3A-based CGBE editors have achieved efficient C-to-G or C-to-A editing at specific preferential loci within the target sites. This advancement presents a new tool for breeding agronomic traits based on SNPs in watermelon.

However, the edited plants obtained using SCGBE2.0 are likely to be C to G/A/T chimeras. For chimeric mutations obtained through SCGBE2.0 editor, the first step is to conduct screening to identify the desired target mutations. This can be achieved through molecular analysis techniques such as sequencing to determine which loci possess the desired mutations. The next step is mutation evaluation, further evaluation is performed on the isolated homozygous individuals, including molecular analysis and phenotypic observations. This helps validate the stability of the mutations and assess their impact on the target traits. Additionally, it is important to consider any unforeseen mutations that may be introduced by chimeric mutations and conduct comprehensive analysis.

In summary, chimeric mutations originating from SCGBE2.0 can be harnessed for the artificial evolution of watermelon genes, with the main goals being the enhancement of specific traits, improvement of gene function, augmented adaptability, or generation of novel functional variations. By engaging in iterative cycles of editing and selection, advantageous mutations can be progressively amassed, thereby facilitating gene evolution.

### Cytosine deaminase substitution may be beneficial to the optimization of SCGBE2.0

Our findings have demonstrated that SCGBE2.0, utilizing hA3A deaminase, enables various forms of base mutations (C-to-G/A) in watermelon. However, the base conversion efficiency and range of SCGBE2.0 can still be further improved due to the limitations of deaminase and Cas protein types. The highest C-to-G conversion efficiency achieved was 46.1% at target position C7, while the highest C-to-A conversion efficiency was 45.9% at position C4. These results indicate that there is room for enhancing the base editing efficiency and expanding the editing scope of SCGBE2.0.

Currently, CGBE research in dicot plants remains relatively limited, with only rAPOBEC1-CGBE being tested [46, 47]. Similar optimization strategies employed in CBE and ABE could potentially be applied to enhance CGBE. These strategies may involve exploring alternative types of deaminases or introducing mutations in key amino acids of deaminases. Such optimizations hold promise for improving CGBE efficiency, adjusting the editing window, and reducing the occurrence of indels. By employing these strategies, further advancements can be made to maximize the potential of CGBE in dicot plants, including watermelon.

### Exploring Cas protein variants presents another avenue for optimizing CGBE in plants

Our experiments using SCGBE2.0, which incorporates nCas9, successfully edited all targets with PAM sequences NGG and NAG. However, no editing was observed for targets with the NGA PAM sequence. The fixed PAM sequences pose a significant limitation to the application of CGBE in plants. To overcome this limitation, the utilization of Cas9 variants, such as SpCas9-VQR (NGA) and ScCas9 (NNG), which have been successfully employed in traditional base editing studies in plants, could expand the range of PAM selections for CGBE [59, 60]. Incorporating these Cas variants into the CGBE system holds potential for broadening the scope of target sites that can be edited. Furthermore, additional optimization of the CGBE vector is still necessary to effectively implement CGBE in watermelon breeding and future applications.

## Conclusions

In conclusion, we conducted a two-step optimization process based on hA3A-CBE to develop a CGBE editor more suitable for watermelon research and molecular breeding. Our results demonstrated that the removal of UGI and the addition of AtUNG to SCBE effectively enhanced the efficiency of C-to-G conversion. The final SCGBE2.0 editor exhibited high editing efficiencies for C-to-G mutations within the C5-C9 editing window, with the highest 46.1% efficiency observed at C7 (Fig. 3g and h). Additionally, SCGBE2.0 achieved highly efficient C-to-A mutations at the C4 position, with an efficiency of up to 45.9% (Fig. 4a and c). Meanwhile, SCGBE2.0 may produce the C-to-G/A/T edited chimeric T0 plants, making it an effective gene editing tool for screening SNPs that affect gene function or for site-directed saturated mutagenesis. However, our data revealed that 28.4% (23/81) of the T0 plants contained varying degrees of indels (Fig. 3b). Therefore, future optimizations of SCGBE2.0 should focus on narrowing the editing window, reducing indel formation, and further increasing base editing efficiency [2, 61]. These improvements will enhance the precision and efficiency of SCGBE2.0 for watermelon genome editing.

## Materials and methods

### Watermelon material

The watermelon material used in this study was ‘TC’ (Tongchuan), which was collected and maintained by our research group. ‘TC’ plants were grown under natural conditions, and mature seeds were collected. Cotyledons were used as explants for watermelon transformation.

### Vector design and plasmid construction

To construct CBE and CGBE vectors, we performed a BLAST search on the *Arabidopsi*s website (Tair: https://www.arabidopsis.org/index.jsp) to obtain the nucleotide sequences of AtUNG (sequence ID: *AT3G18630*), AtUbi (sequence ID: *AT3G52590*), and AtRps5a (sequence ID: *AT3G11940*). The NLS, hA3A, and nCas9 protein sequences were then optimized according to the codon preference of *Arabidopsis* (IDT: https://sg.idtdna.com/pages) and chemically synthesized by Sangon Biotech Co. Ltd (Shanghai, China). The fusion protein sequences were cloned into the pBSE402 vector backbone, which already contained a 35S-GFP expression cassette [62, 63].

#### Construction of CBE vectors

The pBSE402 vector was digested with BamHI/SacI, and the synthetic UGI and NLS fragments were amplified and inserted into the pBSE402 vector to generate UGI-NLS-E9T [64]. After digestion with AscI, the nCas9 fragment was amplified and inserted into UGI-NLS-E9T to obtain the vector: nCas9-UGI-NLS-E9T. Subsequently, the NLS-hAPOBEC3A-nCas9-UGI-NLS-E9T vector was obtained by amplifying and inserting the NLS-hAPOBEC3A fragment into nCas9-UGI-NLS-E9T. Finally, the promoters AtUbi, 2x35S, and AtRps5a were added to produce UCBE, SCBE, and RCBE vectors, respectively.

#### Construction of CGBE1.0 vectors

The pBSE402 vector was digested with BamHI/SacI, and the synthetic nCas9 and NLS fragments were amplified and inserted into the pBSE402 vector to generate nCas9-NLS-E9T. After digestion with AscI, the NLS-hAPOBEC3A fragment was amplified and inserted into nCas9-NLS-E9T to obtain the vector: NLS-hAPOBEC3A-nCas9-NLS-E9T. Finally, the promoters AtUbi and 2x35S were added to the above vector to obtain UCGBE1.0 and SCGBE1.0 vectors, respectively.

#### Construction of SCGBE2.0 vector

The pBSE402 vector was double-digested with BamHI/SacI, and the AtUNG fragment carrying NLS was amplified and inserted into the pBSE402 vector to obtain the vector: UNG-NLS-E9T. After digestion with AscI, the nCas9 fragment was amplified and inserted into UNG-NLS-E9T to obtain the vector: nCas9-UNG-NLS-E9T. Subsequently, the NLS-hAPOBEC3A fragment was amplified and inserted into nCas9-UNG-NLS-E9T to obtain the vector: NLS-hAPOBEC3A-nCas9-UNG-NLS-E9T. Finally, the 2x35S promoter was added to the above vector to obtain the vector: SCGBE2.0.

#### Construction of sgRNA expression plasmids:

For each gene target site, sgRNA expression plasmids were constructed by amplifying the pCBC-DT1T2(Cm) vector and inserting it into the BE vectors after digestion with BsaI. SgRNA1–4 were then assembled into the CBEs, UCGBE1.0, and SCGBE1.0 vectors, respectively, while sgRNA1–7 were assembled into the SCGBE2.0 vector [63].

All PCR fragments for plasmid construction were amplified using Phanta Max 505 Super-Fidelity DNA Polymerase (Vazyme, China). The sequences of all BE vectors and primers used in the construction can be found in Tables S1 and S2 (see online supplementary material), and all vectors are available upon request.

### Transformation of watermelon

Genetic transformation of watermelon was achieved by incubating cotyledons in diluted bacterial medium (final OD600 = 0.08–0.1) for 14 minutes and providing with 40 mg/L acetosyringone. Subsequently, infected cotyledon explants were grown in the dark on MS solid medium containing 1.5 mg/L 6-BA for 3 days. Then, cotyledon fragments were transferred to shoot elongation medium with 200 mg/L Tilmicin and 1.5 mg/L 6-BA, which were kept in normal photoperiod conditions (28°C, 16 h daylight and 8 h dark) for 3 weeks, and transferred to the same fresh medium every 10 days. Plants were screened for successful transformation using a hand-held lamp.

### Genotyping of transgenic watermelon lines

Genomic DNA was extracted from each T0 transgenic callus line using the hexadecyltrimethylammonium bromide (CTAB) method [65]. PCR amplification of the targeted genomic region was performed using specific primers listed in Table S2 (see online supplementary material), and the PCR products were subjected to Sanger sequencing. Additionally, a subset of PCR products with high-quality sequencing data was selected for deep sequencing on the Hi-Tom platform [66], the Hi-TOM strategy streamlines the construction of a multisample hybrid sequencing library to just two common PCR steps. In the initial PCR, target-specific primers with common bridging sequences (5′-ggagtgagtacggtgtgc-3′ and 5′-gagttggatgctggatgg-3′) were used, with the detection site located within 10–100 bp of the primer and an amplification length ranging from 100–300 bp. The first-round PCR products were subsequently barcoded during the second round of PCR. The second-round PCR employed common primers consisting of a platform-specific adaptor sequence, a fixed barcode sequence, and a bridging sequence. Through analysis using the Hi-TOM platform (http://www.hi-tom.net/hi-tom/), detailed information on mutation types and positions of each sample is provided, including read numbers, ratios, mutation types, mutation bases, and DNA sequences. Additionally, the genotype of each sample is analysed and summarized.

## Supplementary Material

Web_Material_uhad155

## Data Availability

All data generated in this study are presented in the paper or the supplementary material. Additional data related to this paper may be requested from the authors.
