# Supplementary material for: Developing a highly efficient CGBE base editor in watermelon
Source: Hortic Res. 2023 Jul 23;10(9):uhad155. doi: 10.1093/hr/uhad155 (PMC10500149; doi:10.1093/hr/uhad155)
Supplement: Web_Material_uhad155 [file web_material_uhad155.docx]

Supplementary figures and tables for

**Developing a highly efficient CGBE editor in watermelon**


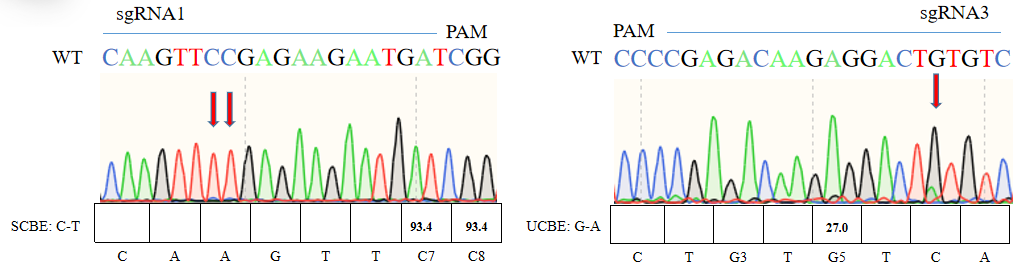


Figure S1: Examples of Sanger sequencing chromatograms for T0 plants with highest C-to-T base editing in sgRNA1 (SCBE) and sgRNA3 (UCBE). The red arrows highlight the editing efficiency of the C-to-T conversion.


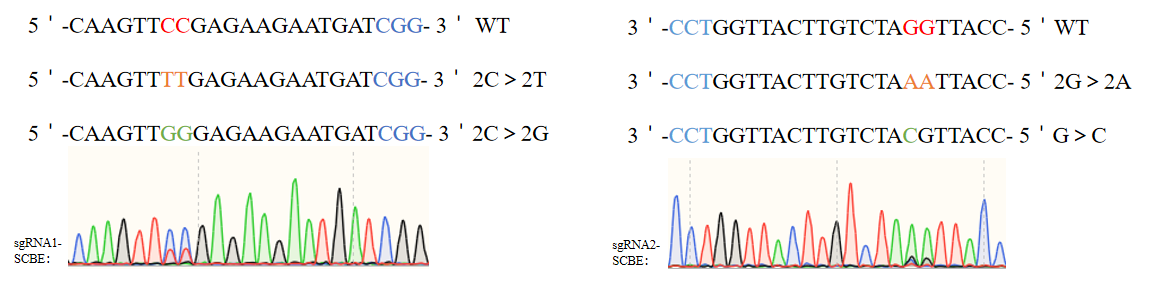


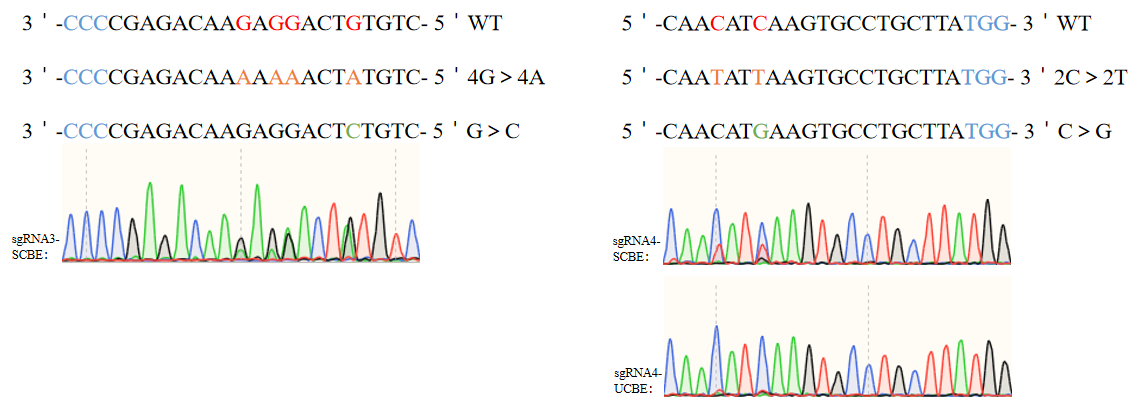


Figure S2: Representative CBE Sanger sequencing chromatograms demonstrating C-to-G edits for four different sgRNAs: sgRNA1-4. The edited bases are highlighted in red (C), orange (T), and green (G), respectively, while the PAM sequence is highlighted in blue.


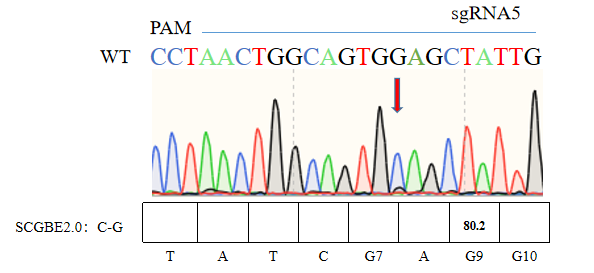


Figure S3: A remarkable sample demonstrating efficient editing at the C9 position of sgRNA5, with an base editing efficiency of 80.2% and no other C sites being edited. The red arrows highlight the editing efficiency of the C-to-G conversion.

**
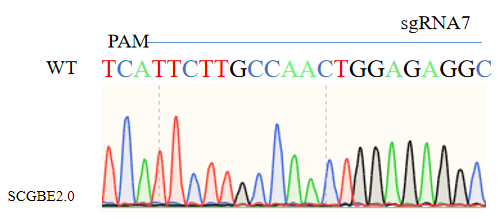
**

Figure S4: Example of a Sanger sequencing chromatogram for T0 plants showing no edits in sgRNA7 by SCGBE2.0 with NGA PAM.

**Table S1 DNA sequences of promoters and vectors**

＞Ubi promoter：

GATCAGGATATTCTTGTTTAAGATGTTGAACTCTATGGAGGTTTGTATGAACTGATGATCTAGGACCGGATAAGTTCCCTTCTTCATAGCGAACTTATTCAAAGAATGTTTTGTGTATCATTCTTGTTACATTGTTATTAATGAAAAAATATTATTGGTCATTGGACTGAACACGAGTGTTAAATATGGACCAGGCCCCAAATAAGATCCATTGATATATGAATTAAATAACAAGAATAAATCGAGTCACCAAACCACTTGCCTTTTTTAACGAGACTTGTTCACCAACTTGATACAAAAGTCATTATCCTATGCAAATCAATAATCATACAAAAATATCCAATAACACTAAAAAATTAAAAGAAATGGATAATTTCACAATATGTTATACGATAAAGAAGTTACTTTTCCAAGAAATTCACTGATTTTATAAGCCCACTTGCATTAGATAAATGGCAAAAAAAAACAAAAAGGAAAAGAAATAAAGCACGAAGAATTCTAGAAAATACGAAATACGCTTCAATGCAGTGGGACCCACGGTTCAATTATTGCCAATTTTCAGCTCCACCGTATATTTAAAAAATAAAACGATAATGCTAAAAAAATATAAATCGTAACGATCGTTAAATCTCAACGGCTGGATCTTATGACGACCGTTAGAAATTGTGGTTGTCGACGAGTCAGTAATAAACGGCGTCAAAGTGGTTGCAGCCGGCACACACGAGTCGTGTTTATCAACTCAAAGCACAAATACTTTTCCTCAACCTAAAAATAAGGCAATTAGCCAAAAACAACTTTGCGTGTAAACAACGCTCAATACACGTGTCATTTTATTATTAGCTATTGCTTCACCGCCTTAGCTTTCTCGTGACCTAGTCGTCCTCGTCTTTTCTTCTTCTTCTTCTATAAAACAATACCCAAAGAGCTCTTCTTCTTCACAATTCAGATTTCAATTTCTCAAAATCTTAAAAACTTTCTCTCAATTCTCTCTACCGTGATCAAGGTAAATTTCTGTGTTCCTTATTCTCTCAAAATCTTCGATTTTGTTTTCGTTCGATCCCAATTTCGTATATGTTCTTTGGTTTAGATTCTGTTAATCTTAGATCGAAGACGATTTTCTGGGTTTGATCGTTAGATATCATCTTAATTCTCGATTAGGGTTTCATAGATATCATCCGATTTGTTCAAATAATTTGAGTTTTGTCGAATAATTACTCTTCGATTTGTGATTTCTATCTAGATCTGGTGTTAGTTTCTAGTTTGTGCGATCGAATTTGTCGATTAATCTGAGTTTTTCTGATTAACAG

＞2×35S promoter：

CGACACTCTCGTCTACTCCAAGAATATCAAAGATACAGTCTCAGAAGACCAAAGGGCTATTGAGACTTTTCAACAAAGGGTAATATCGGGAAACCTCCTCGGATTCCATTGCCCAGCTATCTGTCACTTCATCAAAAGGACAGTAGAAAAGGAAGGTGGCACCTACAAATGCCATCATTGCGATAAAGGAAAGGCTATCGTTCAAGATGCCTCTGCCGACAGTGGTCCCAAAGATGGACCCCCACCCACGAGGAGCATCGTGGAAAAAGAAGACGTTCCAACCACGTCTTCAAAGCAAGTGGATTGATGTGATAACATGGTGGAGCACGACACTCTCGTCTACTCCAAGAATATCAAAGATACAGTCTCAGAAGACCAAAGGGCTATTGAGACTTTTCAACAAAGGGTAATATCGGGAAACCTCCTCGGATTCCATTGCCCAGCTATCTGTCACTTCATCAAAAGGACAGTAGAAAAGGAAGGTGGCACCTACAAATGCCATCATTGCGATAAAGGAAAGGCTATCGTTCAAGATGCCTCTGCCGACAGTGGTCCCAAAGATGGACCCCCACCCACGAGGAGCATCGTGGAAAAAGAAGACGTTCCAACCACGTCTTCAAAGCAAGTGGATTGATGTGATATCTCCACTGACGTAAGGGATGACGCACAATCCCACTATCCTTCGCAAGACCTTCCTCTATATAAGGAAGTTCATTTCATTTGGAGAGGACACGCTGAAATCACCAGTCTCTCTCTACAAATCTATCTCT

>CBE, NLS- APOBEC3A-Linker-nCas9-NLS-UGI-NLS

GATGGCCCCAAAGAAGAAGCGGAAGGTCGGTATCCACGGAGTCCCAGCAGCCGAAGCATCCCCCGCCAGTGGTCCAAGGCATTTGATGGACCCCCATATTTTCACTTCCAACTTTAACAACGGAATTGGACGACATAAAACCTACTTATGCTACGAAGTGGAGAGATTGGACAACGGCACCTCAGTTAAGATGGATCAGCATAGGGGCTTTTTACACAACCAGGCCAAAAACCTCCTGTGCGGGTTTTACGGCCGACACGCCGAATTACGATTCTTGGACCTGGTGCCTTCCTTACAGTTAGACCCTGCGCAAATTTACAGGGTAACTTGGTTCATTTCCTGGAGCCCCTGTTTCAGTTGGGGATGCGCGGGAGAAGTGAGAGCGTTTTTACAAGAGAACACACACGTCAGGCTCCGAATATTTGCCGCTAGAATATACGACTATGACCCGTTATATAAGGAGGCACTGCAAATGCTACGTGACGCAGGCGCCCAAGTAAGTATTATGACATATGACGAGTTCAAACACTGTTGGGATACGTTCGTCGACCACCAAGGATGCCCCTTTCAACCATGGGACGGACTTGACGAGCACAGCCAAGCACTCTCAGGGAGATTAAGGGCCATTCTACAAAACCAGGGCAACTCCGGGAGCGAGACGCCAGGCACCTCCGAGTCGGCCACCCCAGAATCTGACAAGAAGTATTCCATAGGATTGGCTATCGGGACTAACTCAGTTGGATGGGCTGTTATAACGGACGAATACAAAGTACCCAGCAAAAAGTTTAAGGTGTTGGGGAATACCGACCGTCATTCAATCAAGAAAAACCTGATCGGGGCATTACTATTTGACTCAGGGGAGACTGCAGAAGCTACCAGGCTCAAGAGGACTGCCCGTCGTAGATATACTCGTAGAAAAAATCGTATATGTTATCTCCAAGAAATTTTTTCCAACGAAATGGCGAAAGTGGACGACAGTTTCTTTCATAGGCTCGAAGAGTCTTTTCTTGTCGAGGAGGACAAGAAGCATGAAAGGCATCCAATATTTGGCAACATCGTGGACGAGGTTGCTTACCACGAGAAATATCCGACCATATACCATCTTAGAAAAAAACTGGTTGACAGTACGGACAAGGCTGATCTGCGACTAATTTACCTGGCGCTTGCACATATGATTAAATTCAGAGGACACTTCTTAATCGAGGGTGACCTCAACCCGGATAACAGTGACGTCGATAAGTTATTCATCCAATTGGTACAGACTTATAACCAGCTGTTCGAAGAAAATCCAATTAACGCATCCGGTGTCGACGCAAAGGCTATCCTGTCCGCCCGTCTATCCAAGTCTAGGCGTTTGGAAAACCTCATCGCCCAACTCCCTGGGGAAAAGAAGAACGGCCTATTCGGAAATCTAATTGCGCTAAGTCTTGGGCTTACACCTAATTTCAAGAGCAACTTCGACCTAGCAGAGGATGCGAAACTTCAGTTGTCAAAAGATACCTATGACGATGATCTAGACAATCTCTTGGCTCAGATCGGCGATCAATACGCGGACTTGTTCTTGGCGGCAAAGAATCTGTCTGACGCTATTCTACTTTCTGACATTCTTAGGGTTAATACGGAGATTACAAAGGCGCCACTGTCCGCGTCCATGATAAAGAGGTATGATGAGCACCATCAGGACTTAACTCTTCTCAAAGCCTTGGTTCGACAACAGCTCCCAGAGAAGTACAAGGAGATCTTCTTTGACCAGAGCAAAAACGGGTATGCTGGTTACATTGATGGTGGAGCTTCACAGGAGGAGTTCTACAAGTTTATCAAGCCCATCTTGGAGAAAATGGATGGCACTGAGGAGTTGTTAGTTAAACTGAATAGGGAGGACTTGCTGCGAAAACAAAGGACCTTCGATAACGGCAGTATCCCGCACCAAATACACCTGGGTGAGTTGCATGCAATCTTGAGACGACAGGAGGATTTCTATCCGTTTCTTAAAGATAATAGAGAGAAAATAGAAAAAATTCTAACGTTTCGAATACCGTACTACGTGGGACCGCTCGCACGAGGTAACTCCCGTTTCGCTTGGATGACCCGAAAAAGTGAAGAGACTATAACCCCTTGGAACTTTGAAGAAGTTGTCGACAAAGGGGCTTCTGCACAATCTTTTATAGAGAGAATGACCAACTTCGACAAGAACTTGCCAAATGAGAAAGTCTTACCGAAACACAGTCTTTTATACGAGTACTTTACCGTCTACAATGAGTTAACCAAGGTAAAATATGTGACGGAGGGGATGCGAAAACCTGCATTTCTCTCCGGTGAGCAAAAGAAAGCCATTGTGGATCTTCTCTTTAAGACGAATCGTAAAGTAACCGTTAAACAATTGAAAGAAGACTATTTCAAAAAAATTGAGTGTTTTGATTCCGTTGAAATCAGTGGGGTTGAAGATAGGTTCAACGCTTCCTTGGGTACCTACCATGACCTCCTCAAGATTATTAAGGATAAAGATTTTTTAGACAATGAGGAAAATGAGGATATTTTAGAGGACATAGTGCTCACATTGACTCTCTTTGAAGATCGAGAAATGATCGAGGAACGTTTGAAGACATATGCACACCTGTTCGATGATAAAGTCATGAAACAACTAAAACGTCGACGATACACTGGTTGGGGCCGTCTTAGCCGTAAGCTTATCAACGGGATAAGGGACAAGCAGAGTGGTAAAACGATACTCGACTTCCTAAAGAGTGACGGCTTCGCAAACAGAAACTTCATGCAGCTAATTCACGATGATTCATTGACGTTCAAGGAAGATATACAGAAGGCTCAAGTGAGCGGCCAGGGTGACAGCTTGCATGAGCATATAGCCAACCTAGCAGGCTCCCCAGCGATAAAAAAGGGTATTCTTCAAACAGTCAAAGTGGTAGACGAGTTGGTAAAAGTTATGGGGCGACACAAGCCAGAGAATATTGTCATCGAAATGGCGCGAGAAAATCAGACCACGCAAAAGGGGCAGAAAAATAGTAGGGAGCGAATGAAAAGGATAGAAGAGGGCATAAAAGAGTTAGGATCTCAGATACTCAAGGAACATCCGGTGGAAAATACTCAGCTTCAGAATGAAAAACTGTACCTTTATTATCTACAGAATGGGCGAGATATGTATGTAGATCAAGAACTTGATATTAATAGATTGAGTGATTACGACGTTGACCATATAGTCCCACAGTCTTTTCTTAAAGATGACTCCATAGACAATAAGGTCCTTACACGAAGTGACAAAAATAGAGGGAAATCAGACAACGTTCCCAGTGAAGAGGTTGTAAAGAAAATGAAGAATTATTGGAGACAGCTCTTGAATGCAAAACTGATCACTCAAAGAAAATTTGATAATTTGACCAAGGCGGAAAGAGGAGGGTTGAGCGAATTAGACAAAGCCGGTTTCATCAAGAGGCAATTAGTTGAAACAAGACAGATCACCAAGCATGTCGCGCAAATCCTCGACAGTAGGATGAATACGAAGTATGATGAAAATGACAAATTGATCCGTGAGGTAAAGGTCATAACTTTAAAGAGTAAACTGGTAAGCGACTTCAGAAAGGATTTCCAATTTTATAAGGTGAGGGAGATTAATAACTACCACCACGCGCATGACGCGTATCTAAACGCCGTAGTGGGTACCGCTTTGATTAAAAAGTATCCGAAGCTAGAGTCCGAGTTCGTATATGGCGACTATAAGGTATACGATGTAAGGAAAATGATAGCAAAAAGCGAACAGGAGATTGGTAAGGCCACCGCGAAATATTTCTTCTATAGCAATATAATGAATTTCTTCAAGACTGAGATAACCTTGGCGAACGGTGAAATTCGTAAACGTCCCCTGATCGAGACTAATGGAGAGACAGGGGAAATCGTATGGGACAAGGGTAGAGACTTCGCCACCGTTAGGAAAGTACTAAGCATGCCGCAGGTTAATATAGTAAAGAAGACGGAAGTCCAGACGGGGGGCTTCTCAAAAGAATCTATCCTCCCTAAACGAAACTCAGACAAGCTGATTGCCAGAAAGAAAGACTGGGATCCTAAAAAGTACGGGGGGTTTGATTCTCCCACTGTAGCTTATAGTGTACTGGTAGTCGCTAAGGTAGAGAAGGGAAAGTCAAAAAAACTAAAATCTGTGAAGGAACTCTTAGGGATTACCATAATGGAACGTAGTTCATTCGAGAAGAATCCTATAGATTTTTTGGAAGCTAAGGGGTATAAGGAAGTCAAAAAAGATCTCATTATAAAGTTGCCAAAATATTCACTCTTCGAACTGGAGAATGGGCGTAAGCGTATGCTTGCATCCGCTGGTGAGCTGCAGAAGGGCAATGAATTAGCCCTGCCATCCAAATATGTAAATTTCCTATACTTAGCTTCTCACTATGAGAAATTGAAGGGCAGCCCTGAAGACAATGAGCAAAAACAGCTGTTTGTCGAACAACACAAACACTACCTTGACGAGATTATCGAGCAGATAAGTGAGTTCTCCAAACGAGTCATATTAGCGGATGCGAACTTAGACAAGGTTCTGAGCGCGTACAACAAGCATCGTGACAAGCCCATTCGAGAGCAGGCTGAAAATATTATCCATCTGTTTACGCTAACGAACCTAGGAGCGCCAGCTGCATTCAAATATTTCGATACGACGATTGACAGGAAAAGATACACCTCAACCAAAGAGGTCTTGGACGCGACGCTAATACATCAGTCTATCACGGGACTATACGAGACGCGAATCGATCTGTCTCAACTTGGTGGCGATAAGAGGCCAGCGGCGACGAAAAAAGCTGGACAGGCCAAGAAGAAGAAAGAATTTTCCGGAGGGAGTACGAATCTCAGTGACATAATTGAGAAAGAAACGGGAAAGCAATTAGTTATTCAGGAGTCAATTTTGATGCTCCCAGAGGAGGTAGAAGAAGTTATAGGGAACAAACCTGAATCAGACATTTTAGTGCACACGGCGTATGACGAGTCAACTGATGAAAATGTGATGTTACTGACGTCAGACGCTCCAGAGTATAAACCTTGGGCGCTAGTGATACAGGACAGCAACGGCGAGAATAAAATTAAGATGTTATCTGGCGGAAGTCCTAAGAAGAAGAGGAAGGTATAG

>CGBE1.0, NLS- APOBEC3A-Linker-nCas9-NLS

GATGGCCCCAAAGAAGAAGCGGAAGGTCGGTATCCACGGAGTCCCAGCAGCCGAAGCATCCCCCGCCAGTGGTCCAAGGCATTTGATGGACCCCCATATTTTCACTTCCAACTTTAACAACGGAATTGGACGACATAAAACCTACTTATGCTACGAAGTGGAGAGATTGGACAACGGCACCTCAGTTAAGATGGATCAGCATAGGGGCTTTTTACACAACCAGGCCAAAAACCTCCTGTGCGGGTTTTACGGCCGACACGCCGAATTACGATTCTTGGACCTGGTGCCTTCCTTACAGTTAGACCCTGCGCAAATTTACAGGGTAACTTGGTTCATTTCCTGGAGCCCCTGTTTCAGTTGGGGATGCGCGGGAGAAGTGAGAGCGTTTTTACAAGAGAACACACACGTCAGGCTCCGAATATTTGCCGCTAGAATATACGACTATGACCCGTTATATAAGGAGGCACTGCAAATGCTACGTGACGCAGGCGCCCAAGTAAGTATTATGACATATGACGAGTTCAAACACTGTTGGGATACGTTCGTCGACCACCAAGGATGCCCCTTTCAACCATGGGACGGACTTGACGAGCACAGCCAAGCACTCTCAGGGAGATTAAGGGCCATTCTACAAAACCAGGGCAACTCCGGGAGCGAGACGCCAGGCACCTCCGAGTCGGCCACCCCAGAATCTGACAAGAAGTATTCCATAGGATTGGCTATCGGGACTAACTCAGTTGGATGGGCTGTTATAACGGACGAATACAAAGTACCCAGCAAAAAGTTTAAGGTGTTGGGGAATACCGACCGTCATTCAATCAAGAAAAACCTGATCGGGGCATTACTATTTGACTCAGGGGAGACTGCAGAAGCTACCAGGCTCAAGAGGACTGCCCGTCGTAGATATACTCGTAGAAAAAATCGTATATGTTATCTCCAAGAAATTTTTTCCAACGAAATGGCGAAAGTGGACGACAGTTTCTTTCATAGGCTCGAAGAGTCTTTTCTTGTCGAGGAGGACAAGAAGCATGAAAGGCATCCAATATTTGGCAACATCGTGGACGAGGTTGCTTACCACGAGAAATATCCGACCATATACCATCTTAGAAAAAAACTGGTTGACAGTACGGACAAGGCTGATCTGCGACTAATTTACCTGGCGCTTGCACATATGATTAAATTCAGAGGACACTTCTTAATCGAGGGTGACCTCAACCCGGATAACAGTGACGTCGATAAGTTATTCATCCAATTGGTACAGACTTATAACCAGCTGTTCGAAGAAAATCCAATTAACGCATCCGGTGTCGACGCAAAGGCTATCCTGTCCGCCCGTCTATCCAAGTCTAGGCGTTTGGAAAACCTCATCGCCCAACTCCCTGGGGAAAAGAAGAACGGCCTATTCGGAAATCTAATTGCGCTAAGTCTTGGGCTTACACCTAATTTCAAGAGCAACTTCGACCTAGCAGAGGATGCGAAACTTCAGTTGTCAAAAGATACCTATGACGATGATCTAGACAATCTCTTGGCTCAGATCGGCGATCAATACGCGGACTTGTTCTTGGCGGCAAAGAATCTGTCTGACGCTATTCTACTTTCTGACATTCTTAGGGTTAATACGGAGATTACAAAGGCGCCACTGTCCGCGTCCATGATAAAGAGGTATGATGAGCACCATCAGGACTTAACTCTTCTCAAAGCCTTGGTTCGACAACAGCTCCCAGAGAAGTACAAGGAGATCTTCTTTGACCAGAGCAAAAACGGGTATGCTGGTTACATTGATGGTGGAGCTTCACAGGAGGAGTTCTACAAGTTTATCAAGCCCATCTTGGAGAAAATGGATGGCACTGAGGAGTTGTTAGTTAAACTGAATAGGGAGGACTTGCTGCGAAAACAAAGGACCTTCGATAACGGCAGTATCCCGCACCAAATACACCTGGGTGAGTTGCATGCAATCTTGAGACGACAGGAGGATTTCTATCCGTTTCTTAAAGATAATAGAGAGAAAATAGAAAAAATTCTAACGTTTCGAATACCGTACTACGTGGGACCGCTCGCACGAGGTAACTCCCGTTTCGCTTGGATGACCCGAAAAAGTGAAGAGACTATAACCCCTTGGAACTTTGAAGAAGTTGTCGACAAAGGGGCTTCTGCACAATCTTTTATAGAGAGAATGACCAACTTCGACAAGAACTTGCCAAATGAGAAAGTCTTACCGAAACACAGTCTTTTATACGAGTACTTTACCGTCTACAATGAGTTAACCAAGGTAAAATATGTGACGGAGGGGATGCGAAAACCTGCATTTCTCTCCGGTGAGCAAAAGAAAGCCATTGTGGATCTTCTCTTTAAGACGAATCGTAAAGTAACCGTTAAACAATTGAAAGAAGACTATTTCAAAAAAATTGAGTGTTTTGATTCCGTTGAAATCAGTGGGGTTGAAGATAGGTTCAACGCTTCCTTGGGTACCTACCATGACCTCCTCAAGATTATTAAGGATAAAGATTTTTTAGACAATGAGGAAAATGAGGATATTTTAGAGGACATAGTGCTCACATTGACTCTCTTTGAAGATCGAGAAATGATCGAGGAACGTTTGAAGACATATGCACACCTGTTCGATGATAAAGTCATGAAACAACTAAAACGTCGACGATACACTGGTTGGGGCCGTCTTAGCCGTAAGCTTATCAACGGGATAAGGGACAAGCAGAGTGGTAAAACGATACTCGACTTCCTAAAGAGTGACGGCTTCGCAAACAGAAACTTCATGCAGCTAATTCACGATGATTCATTGACGTTCAAGGAAGATATACAGAAGGCTCAAGTGAGCGGCCAGGGTGACAGCTTGCATGAGCATATAGCCAACCTAGCAGGCTCCCCAGCGATAAAAAAGGGTATTCTTCAAACAGTCAAAGTGGTAGACGAGTTGGTAAAAGTTATGGGGCGACACAAGCCAGAGAATATTGTCATCGAAATGGCGCGAGAAAATCAGACCACGCAAAAGGGGCAGAAAAATAGTAGGGAGCGAATGAAAAGGATAGAAGAGGGCATAAAAGAGTTAGGATCTCAGATACTCAAGGAACATCCGGTGGAAAATACTCAGCTTCAGAATGAAAAACTGTACCTTTATTATCTACAGAATGGGCGAGATATGTATGTAGATCAAGAACTTGATATTAATAGATTGAGTGATTACGACGTTGACCATATAGTCCCACAGTCTTTTCTTAAAGATGACTCCATAGACAATAAGGTCCTTACACGAAGTGACAAAAATAGAGGGAAATCAGACAACGTTCCCAGTGAAGAGGTTGTAAAGAAAATGAAGAATTATTGGAGACAGCTCTTGAATGCAAAACTGATCACTCAAAGAAAATTTGATAATTTGACCAAGGCGGAAAGAGGAGGGTTGAGCGAATTAGACAAAGCCGGTTTCATCAAGAGGCAATTAGTTGAAACAAGACAGATCACCAAGCATGTCGCGCAAATCCTCGACAGTAGGATGAATACGAAGTATGATGAAAATGACAAATTGATCCGTGAGGTAAAGGTCATAACTTTAAAGAGTAAACTGGTAAGCGACTTCAGAAAGGATTTCCAATTTTATAAGGTGAGGGAGATTAATAACTACCACCACGCGCATGACGCGTATCTAAACGCCGTAGTGGGTACCGCTTTGATTAAAAAGTATCCGAAGCTAGAGTCCGAGTTCGTATATGGCGACTATAAGGTATACGATGTAAGGAAAATGATAGCAAAAAGCGAACAGGAGATTGGTAAGGCCACCGCGAAATATTTCTTCTATAGCAATATAATGAATTTCTTCAAGACTGAGATAACCTTGGCGAACGGTGAAATTCGTAAACGTCCCCTGATCGAGACTAATGGAGAGACAGGGGAAATCGTATGGGACAAGGGTAGAGACTTCGCCACCGTTAGGAAAGTACTAAGCATGCCGCAGGTTAATATAGTAAAGAAGACGGAAGTCCAGACGGGGGGCTTCTCAAAAGAATCTATCCTCCCTAAACGAAACTCAGACAAGCTGATTGCCAGAAAGAAAGACTGGGATCCTAAAAAGTACGGGGGGTTTGATTCTCCCACTGTAGCTTATAGTGTACTGGTAGTCGCTAAGGTAGAGAAGGGAAAGTCAAAAAAACTAAAATCTGTGAAGGAACTCTTAGGGATTACCATAATGGAACGTAGTTCATTCGAGAAGAATCCTATAGATTTTTTGGAAGCTAAGGGGTATAAGGAAGTCAAAAAAGATCTCATTATAAAGTTGCCAAAATATTCACTCTTCGAACTGGAGAATGGGCGTAAGCGTATGCTTGCATCCGCTGGTGAGCTGCAGAAGGGCAATGAATTAGCCCTGCCATCCAAATATGTAAATTTCCTATACTTAGCTTCTCACTATGAGAAATTGAAGGGCAGCCCTGAAGACAATGAGCAAAAACAGCTGTTTGTCGAACAACACAAACACTACCTTGACGAGATTATCGAGCAGATAAGTGAGTTCTCCAAACGAGTCATATTAGCGGATGCGAACTTAGACAAGGTTCTGAGCGCGTACAACAAGCATCGTGACAAGCCCATTCGAGAGCAGGCTGAAAATATTATCCATCTGTTTACGCTAACGAACCTAGGAGCGCCAGCTGCATTCAAATATTTCGATACGACGATTGACAGGAAAAGATACACCTCAACCAAAGAGGTCTTGGACGCGACGCTAATACATCAGTCTATCACGGGACTATACGAGACGCGAATCGATCTGTCTCAACTTGGTGGCGATTCTGGCGGAAGTCCTAAGAAGAAGAGGAAGGTATAG

>CGBE2.0, NLS- APOBEC3A-Linker-nCas9-NLS-AtUNG-NLS

GATGGCCCCAAAGAAGAAGCGGAAGGTCGGTATCCACGGAGTCCCAGCAGCCGAAGCATCCCCCGCCAGTGGTCCAAGGCATTTGATGGACCCCCATATTTTCACTTCCAACTTTAACAACGGAATTGGACGACATAAAACCTACTTATGCTACGAAGTGGAGAGATTGGACAACGGCACCTCAGTTAAGATGGATCAGCATAGGGGCTTTTTACACAACCAGGCCAAAAACCTCCTGTGCGGGTTTTACGGCCGACACGCCGAATTACGATTCTTGGACCTGGTGCCTTCCTTACAGTTAGACCCTGCGCAAATTTACAGGGTAACTTGGTTCATTTCCTGGAGCCCCTGTTTCAGTTGGGGATGCGCGGGAGAAGTGAGAGCGTTTTTACAAGAGAACACACACGTCAGGCTCCGAATATTTGCCGCTAGAATATACGACTATGACCCGTTATATAAGGAGGCACTGCAAATGCTACGTGACGCAGGCGCCCAAGTAAGTATTATGACATATGACGAGTTCAAACACTGTTGGGATACGTTCGTCGACCACCAAGGATGCCCCTTTCAACCATGGGACGGACTTGACGAGCACAGCCAAGCACTCTCAGGGAGATTAAGGGCCATTCTACAAAACCAGGGCAACTCCGGGAGCGAGACGCCAGGCACCTCCGAGTCGGCCACCCCAGAATCTGACAAGAAGTATTCCATAGGATTGGCTATCGGGACTAACTCAGTTGGATGGGCTGTTATAACGGACGAATACAAAGTACCCAGCAAAAAGTTTAAGGTGTTGGGGAATACCGACCGTCATTCAATCAAGAAAAACCTGATCGGGGCATTACTATTTGACTCAGGGGAGACTGCAGAAGCTACCAGGCTCAAGAGGACTGCCCGTCGTAGATATACTCGTAGAAAAAATCGTATATGTTATCTCCAAGAAATTTTTTCCAACGAAATGGCGAAAGTGGACGACAGTTTCTTTCATAGGCTCGAAGAGTCTTTTCTTGTCGAGGAGGACAAGAAGCATGAAAGGCATCCAATATTTGGCAACATCGTGGACGAGGTTGCTTACCACGAGAAATATCCGACCATATACCATCTTAGAAAAAAACTGGTTGACAGTACGGACAAGGCTGATCTGCGACTAATTTACCTGGCGCTTGCACATATGATTAAATTCAGAGGACACTTCTTAATCGAGGGTGACCTCAACCCGGATAACAGTGACGTCGATAAGTTATTCATCCAATTGGTACAGACTTATAACCAGCTGTTCGAAGAAAATCCAATTAACGCATCCGGTGTCGACGCAAAGGCTATCCTGTCCGCCCGTCTATCCAAGTCTAGGCGTTTGGAAAACCTCATCGCCCAACTCCCTGGGGAAAAGAAGAACGGCCTATTCGGAAATCTAATTGCGCTAAGTCTTGGGCTTACACCTAATTTCAAGAGCAACTTCGACCTAGCAGAGGATGCGAAACTTCAGTTGTCAAAAGATACCTATGACGATGATCTAGACAATCTCTTGGCTCAGATCGGCGATCAATACGCGGACTTGTTCTTGGCGGCAAAGAATCTGTCTGACGCTATTCTACTTTCTGACATTCTTAGGGTTAATACGGAGATTACAAAGGCGCCACTGTCCGCGTCCATGATAAAGAGGTATGATGAGCACCATCAGGACTTAACTCTTCTCAAAGCCTTGGTTCGACAACAGCTCCCAGAGAAGTACAAGGAGATCTTCTTTGACCAGAGCAAAAACGGGTATGCTGGTTACATTGATGGTGGAGCTTCACAGGAGGAGTTCTACAAGTTTATCAAGCCCATCTTGGAGAAAATGGATGGCACTGAGGAGTTGTTAGTTAAACTGAATAGGGAGGACTTGCTGCGAAAACAAAGGACCTTCGATAACGGCAGTATCCCGCACCAAATACACCTGGGTGAGTTGCATGCAATCTTGAGACGACAGGAGGATTTCTATCCGTTTCTTAAAGATAATAGAGAGAAAATAGAAAAAATTCTAACGTTTCGAATACCGTACTACGTGGGACCGCTCGCACGAGGTAACTCCCGTTTCGCTTGGATGACCCGAAAAAGTGAAGAGACTATAACCCCTTGGAACTTTGAAGAAGTTGTCGACAAAGGGGCTTCTGCACAATCTTTTATAGAGAGAATGACCAACTTCGACAAGAACTTGCCAAATGAGAAAGTCTTACCGAAACACAGTCTTTTATACGAGTACTTTACCGTCTACAATGAGTTAACCAAGGTAAAATATGTGACGGAGGGGATGCGAAAACCTGCATTTCTCTCCGGTGAGCAAAAGAAAGCCATTGTGGATCTTCTCTTTAAGACGAATCGTAAAGTAACCGTTAAACAATTGAAAGAAGACTATTTCAAAAAAATTGAGTGTTTTGATTCCGTTGAAATCAGTGGGGTTGAAGATAGGTTCAACGCTTCCTTGGGTACCTACCATGACCTCCTCAAGATTATTAAGGATAAAGATTTTTTAGACAATGAGGAAAATGAGGATATTTTAGAGGACATAGTGCTCACATTGACTCTCTTTGAAGATCGAGAAATGATCGAGGAACGTTTGAAGACATATGCACACCTGTTCGATGATAAAGTCATGAAACAACTAAAACGTCGACGATACACTGGTTGGGGCCGTCTTAGCCGTAAGCTTATCAACGGGATAAGGGACAAGCAGAGTGGTAAAACGATACTCGACTTCCTAAAGAGTGACGGCTTCGCAAACAGAAACTTCATGCAGCTAATTCACGATGATTCATTGACGTTCAAGGAAGATATACAGAAGGCTCAAGTGAGCGGCCAGGGTGACAGCTTGCATGAGCATATAGCCAACCTAGCAGGCTCCCCAGCGATAAAAAAGGGTATTCTTCAAACAGTCAAAGTGGTAGACGAGTTGGTAAAAGTTATGGGGCGACACAAGCCAGAGAATATTGTCATCGAAATGGCGCGAGAAAATCAGACCACGCAAAAGGGGCAGAAAAATAGTAGGGAGCGAATGAAAAGGATAGAAGAGGGCATAAAAGAGTTAGGATCTCAGATACTCAAGGAACATCCGGTGGAAAATACTCAGCTTCAGAATGAAAAACTGTACCTTTATTATCTACAGAATGGGCGAGATATGTATGTAGATCAAGAACTTGATATTAATAGATTGAGTGATTACGACGTTGACCATATAGTCCCACAGTCTTTTCTTAAAGATGACTCCATAGACAATAAGGTCCTTACACGAAGTGACAAAAATAGAGGGAAATCAGACAACGTTCCCAGTGAAGAGGTTGTAAAGAAAATGAAGAATTATTGGAGACAGCTCTTGAATGCAAAACTGATCACTCAAAGAAAATTTGATAATTTGACCAAGGCGGAAAGAGGAGGGTTGAGCGAATTAGACAAAGCCGGTTTCATCAAGAGGCAATTAGTTGAAACAAGACAGATCACCAAGCATGTCGCGCAAATCCTCGACAGTAGGATGAATACGAAGTATGATGAAAATGACAAATTGATCCGTGAGGTAAAGGTCATAACTTTAAAGAGTAAACTGGTAAGCGACTTCAGAAAGGATTTCCAATTTTATAAGGTGAGGGAGATTAATAACTACCACCACGCGCATGACGCGTATCTAAACGCCGTAGTGGGTACCGCTTTGATTAAAAAGTATCCGAAGCTAGAGTCCGAGTTCGTATATGGCGACTATAAGGTATACGATGTAAGGAAAATGATAGCAAAAAGCGAACAGGAGATTGGTAAGGCCACCGCGAAATATTTCTTCTATAGCAATATAATGAATTTCTTCAAGACTGAGATAACCTTGGCGAACGGTGAAATTCGTAAACGTCCCCTGATCGAGACTAATGGAGAGACAGGGGAAATCGTATGGGACAAGGGTAGAGACTTCGCCACCGTTAGGAAAGTACTAAGCATGCCGCAGGTTAATATAGTAAAGAAGACGGAAGTCCAGACGGGGGGCTTCTCAAAAGAATCTATCCTCCCTAAACGAAACTCAGACAAGCTGATTGCCAGAAAGAAAGACTGGGATCCTAAAAAGTACGGGGGGTTTGATTCTCCCACTGTAGCTTATAGTGTACTGGTAGTCGCTAAGGTAGAGAAGGGAAAGTCAAAAAAACTAAAATCTGTGAAGGAACTCTTAGGGATTACCATAATGGAACGTAGTTCATTCGAGAAGAATCCTATAGATTTTTTGGAAGCTAAGGGGTATAAGGAAGTCAAAAAAGATCTCATTATAAAGTTGCCAAAATATTCACTCTTCGAACTGGAGAATGGGCGTAAGCGTATGCTTGCATCCGCTGGTGAGCTGCAGAAGGGCAATGAATTAGCCCTGCCATCCAAATATGTAAATTTCCTATACTTAGCTTCTCACTATGAGAAATTGAAGGGCAGCCCTGAAGACAATGAGCAAAAACAGCTGTTTGTCGAACAACACAAACACTACCTTGACGAGATTATCGAGCAGATAAGTGAGTTCTCCAAACGAGTCATATTAGCGGATGCGAACTTAGACAAGGTTCTGAGCGCGTACAACAAGCATCGTGACAAGCCCATTCGAGAGCAGGCTGAAAATATTATCCATCTGTTTACGCTAACGAACCTAGGAGCGCCAGCTGCATTCAAATATTTCGATACGACGATTGACAGGAAAAGATACACCTCAACCAAAGAGGTCTTGGACGCGACGCTAATACATCAGTCTATCACGGGACTATACGAGACGCGAATCGATCTGTCTCAACTTGGTGGCGATAAGAGGCCAGCGGCGACGAAAAAAGCTGGACAGGCCAAGAAGAAGAAAGAATTTTCCGGAGGGAGTGCTTCGTCGACACCTAAAACCCTAATGGATTTTTTTCAACCTGCCAAACGCCTCAAAGCTTCCCCTTCTTCCTCCTCTTTCCCTGCCGTCTCCGTCGCCGGTGGTTCCCGTGATTTGGGTTCTGTAGCAAACTCGCCGCCTCGTGTAACCGTTACCACTTCCGTCGCCGATGATTCGTCTGGCCTTACACCTGAACAGATCGCTCGCGCAGAGTTCAACAAGTTCGTCGCCAAGTCCAAGCGTAACCTCGCCGTCTGCTCCGAGAGGGTCACAAAAGCAAAATCTGAAGGAAACTGCTACGTACCATTGAGTGAGCTCTTAGTAGAAGAATCATGGCTTAAAGCTCTTCCTGGGGAATTTCATAAACCCTACGCCAAATCACTTTCTGATTTCCTTGAACGTGAGATCATCACTGACAGTAAAAGCCCTCTGATTTATCCACCGCAGCACTTGATTTTCAATGCTCTTAATACAACTCCTTTTGATCGAGTTAAGACTGTCATTATCGGACAGGATCCTTATCATGGACCTGGTCAAGCTATGGGTTTGTCCTTCTCTGTACCTGAAGGAGAAAAGCTTCCTTCTAGTCTGTTGAACATCTTTAAGGAGCTTCATAAAGATGTTGGCTGTTCCATCCCACGTCACGGTAATCTACAGAAATGGGCTGTGCAGGGTGTGTTACTCCTGAATGCTGTTCTTACAGTAAGGAGTAAACAGCCTAATTCACATGCAAAGAAAGGATGGGAACAATTCACTGATGCTGTTATTCAAAGTATCTCACAGCAGAAGGAAGGTGTTGTTTTTCTTCTCTGGGGAAGATACGCTCAAGAGAAATCCAAGTTGATAGATGCGACTAAACATCATATACTCACAGCAGCTCATCCATCTGGTTTGTCGGCGAATAGAGGCTTCTTCGACTGCAGGCATTTCTCTCGCGCAAACCAGCTACTCGAGGAAATGGGGATTCCTCCCATAGACTGGCAACTTTCTGGCGGAAGTCCTAAGAAGAAGAGGAAGGTATAG

**Supplemental Table 2. Primers used in this study**

| Name | Sequence (5’→3’) | Application |
| --- | --- | --- |
| sgRNA1/2F | CCGCTCTTCAATTATCCGTAACG | PCR for Sanger sequencing of sgRNA1/2 target site |
| sgRNA1/2R | CAATCTCAGCCGAATCAATATCG |  |
| sgRNA3F | GTCGAAAGGCAAGCaCTAAA | PCR for Sanger sequencing of sgRNA3 target site |
| sgRNA3R | CCGACATCGTGCTCGATACT |  |
| sgRNA4F | AGTACATTATGTTGACACCGG | PCR for Sanger sequencing of sgRNA4 target site |
| sgRNA4R | AGATACTCCAAATTTGTAACAT |  |
| sgRNA5F | GAGTAGGTTGGTCTGATGAATCT | PCR for Sanger sequencing of sgRNA5 target site |
| sgRNA5R | CCGACTATCCACAACAACCC |  |
| sgRNA6F | AACATGGTATCAGAGCAAGTGG | PCR for Sanger sequencing of sgRNA6 target site |
| sgRNA6R | GATGGCCATTGGTCTGTCTCC |  |
| 1-F | ggagtgagtacggtgtgcGCATTGCAACTTCCGGCC | PCR for Hi-TOM sequencing of sgRNA1 target site |
| 1-R | gagttggatgctggatggGCCTCGCTCACAATCCTAG |  |
| 2-F | ggagtgagtacggtgtgcGGTCGACCTGGTCCTGTT | PCR for Hi-TOM sequencing of sgRNA2 target site |
| 2-R | gagttggatgctggatggCACCCTCCACCCACGTAA |  |
| 3-F | ggagtgagtacggtgtgcGTACTGTGAATCTGTGTTGA | PCR for Hi-TOM sequencing of sgRNA3 target site |
| 3-R | gagttggatgctggatggGATCAATGAACATCTTGATTC |  |
| 4-F | ggagtgagtacggtgtgcAGTACATTATGTTGACACCGG | PCR for Hi-TOM sequencing of sgRNA4 target site |
| 4-R | gagttggatgctggatggAGATACTCCAAATTTGTAACAT |  |
| 5-F | ggagtgagtacggtgtgcCAGATGCCTAATGGAGAAGC | PCR for Hi-TOM sequencing of sgRNA5 target site |
| 5-R | gagttggatgctggatggAATTATAGGCTGATCAAGCC |  |
| 6-F | ggagtgagtacggtgtgcGCCAAGTCCCTCATGAATGT | PCR for Hi-TOM sequencing of sgRNA6 target site |
| 6-R | gagttggatgctggatggCCTTCAACTTTCATCAGCTC |  |
| UNG-F | taaaccaataagcttgcatgGGCGCGCCGCTTCGTCGACACCTAAAAC | Primers for vector construction |
| UNG-R | tacgaacgaaagctctCCTGCAGGCTATACCTTCCTCTTCTTCTTAGGACTTCCGCCAGAAAGTTGCCAGTCTATGGGA |  |
| nCas9-F | aaaccaataagcttgcatgGGCGCGCCGACAAGAAGTATTCCATAGG |  |
| nCas9-R | TTTTAGGTGTCGACGAAGCACTCCCTCCGGAAAATTCTT |  |
| hA3A-F | aaaccaataagcttgcatgGGCGCGCCgatggccccaaagaagaagc |  |
| hA3A-R | CCTATGGAATACTTCTTGTCAGATTCTGGGGTGGCCGA |  |
| 35S-F | taaaccaataagcttgcatgGGCGCGCCcagctgcattaatgaatcgg |  |
| 35S-R | cttcttctttggggccatcctcgagagagatagatttgtaga |  |
| Ubi-F | aacGGTTACCTCTAGActagtgatcaggatattcttgtt |  |
| Ubi-R | cttcttctttggggccatcctgttaatcagaaaaactca |  |
